# 

**Published:** 1886-04-20

**Authors:** 


### EDITORIALS AND MISCELLANEOUS.

### EDITORIAL NOTICES.

CHOLERA is still prevailing in Brittany, a western province of France. It may yet reach America.

THE FARE to the Ameriran Medical Association, on the various Railroads, is one regular fare and a third, instead of one-third as formerly reported.

COCAINE.—This drug is now quoted at $15 per ounce—a large decline from former rates. Our druggists generally retail the article at 10 cents a grain.

SANITARY CONVENTION.—The Pennsylvania State Board of Health are arranging for a National Sanitary Convention, to be held at Philadelphia in the month of May.

DAILY MEDICAL JOURNAL.—There is but one daily medical journal, it is said, in the world—entitled The Medical Reform (La Reform a Medica), and published in Naples.

FOR SALE.—A Scholarship for a complete Course of Instruction in Bryant and Stratton's Business College, Louisville, Ky. Apply to R. C. WORD, M.D., Editor of this journal, Atlanta, Ga.

CHEAP CHLOROFORM.—A process for manufacturing this article without the use of alcohol has been discovered, by which it has been greatly reduced in price. It is now said to be procurable in large lots at 40 cents per pound.

ALCOHOL WEAKENING.—The Internal Revenue receipts for the last year, is reported at $9,000,000 less than the previous year—showing a great fall-off in the consumption of alcohol in this country. Push on the ball, say we.

BALTIMORE publishes four medical journals ; has two medical colleges, twenty-two regular professors, and twenty-one lecturers, aggregating forty-three teachers. In addition to these, she claims to have four medical societies, etc. Too much physic, we fear.

SCARLETT WRAPPERS are required upon bottles of Morphine in the State of Georgia ; also in Florida and Kentucky. It should be done everywhere, or, what would be still better, something should be added in the manufacture of the article that would change its color, so that it could be easily distinguished from quinine.

ABDOMINAL SURGERY.—We learn that the subject of Abdominal Surgery will be made a conspicuous feature of the approaching meeting of the American Medical Association. It is understood that a number of able papers upon the subject will be read, and it is expected that the discussions which they will provoke will be both interesting and instructive.

LONGEVITY.—The DeKalb Chronicle, a newspaper printed at Decatur, the county-site of DeKalb County, Ga.—a village six miles East of Atlanta—publishes the names of one hundred citizens, all of whom are over seventy-five years of age ; and a number over ninety—running as high as ninety-seven years—resident in the vicinity of that village.

BRYANT & STRATTON'S BUSINESS COLLEGE, LOUISVILLE, KY.—This is a good place to give your son a business course which will prepare him to enter at once into active life, either as book-keeper or in any other capacity connected with mercantile or other lines of business. See their advertisement in another part of this journal. See also, special notice, of a Scholarship for sale.

HEMICRANIA.—Dr. E. van Goidtsnoven writes us that he has relieved a case of violent hemicrania by the hypodermic injection of fifteen drops of a 4-percent. solution of cocaine, at three points above the brow on the affected side—five drops at each point. The relief was almost instantaneous, and the pain did not return. It would doubtless relieve facial neuralgia with equal promptness, and, probably also, nervous or sick-headache.

DEATH OF DR. AUSTIN FLINT.—This eminent man in the profession died of apoplexy in New York City, on the 13th of March, 1886, aged seventy-three years. He is prominently known throughout the medical world, having occupied numerous positions of importance. Since 1861 he has been the Professor of the Principles and Practice of Medicine in the Bellevue Hospital Medical College. He has been an author of distinction—was a few years ago president of the American Medical Association, and was to have been the president of the International Medical Congress to meet in Washington, D. C., in 1887.

THE GEORGIA MEDICAL ASSOCIATION.—Remember that the State Medieal Association will meet the present year, on the 21st of April, in the beautiful city of Augusta. As stated in our last, a large meeting is anticipated, and it will doubtless be a pleasant one, as the profession of Augusta are noted for Intelligence, sociability, and generous hospitality.

We are informed by Dr. Goodrich, chairman of the committee of arrangements, that the Railroads will furnish tickets to members at four cents per mile, round trip, and that the hotels will take members as follows : Planters' Hotel, $2.50 per day ; Globe and Central Hotels, each, $2 per day ; Augusta Hotel, $1.50 per day ; Adkins House, $1 per day.

